# Electrocatalytical Nitrite Oxidation via Manganese and Copper Oxides on Carbon Screen-Printed Electrode

**DOI:** 10.3390/s25123764

**Published:** 2025-06-16

**Authors:** Roberta Farina, Silvia Scalese, Alessandra Alberti, Stefania Maria Serena Privitera, Giuseppe Emanuele Capuano, Domenico Corso, Giuseppe Andrea Screpis, Serena Concetta Rita Reina, Guglielmo Guido Condorelli, Maria Anna Coniglio, Sebania Libertino

**Affiliations:** 1 Consiglio Nazionale delle Ricerche—Istituto per la Microelettronica e Microsistemi (CNR-IMM), Strada VIII Z.I., 5, 95121 Catania, Italy; 2 Dipartimento di Scienze Chimiche, Università Degli Studi di Catania, Viale A. Doria 6, 95125 Catania, Italy; 3 Department of Electrical, Computer and Biomedical Engineering, Università Degli Studi di Pavia, Via Ferrata 5, 27100 Pavia, Italy; 4 Dipartimento di Scienze Chimiche, Biologiche, Farmaceutiche e Analitiche (ChiBioFarAm), Università Degli Studi di Messina, Viale F. Stagno d’Alcontres 31, Vill. S. Agata, 98166 Messina, Italy; 5 Consiglio Nazionale delle Ricerche—Istituto per lo Studio dei Materiali Nanostrutturati, CNR-ISMN, Viale F. Stagno d’Alcontres 31, Vill. S. Agata, 98166 Messina, Italy; 6 Dipartimento di Scienze Mediche, Chirurgiche e Tecnologie Avanzate “G.F. Ingrassia”, Università Degli Studi di Catania, Via S. Sofia 87, 95123 Catania, Italy

**Keywords:** nitrite sensing, metal oxides, electrocatalysis, electrodeposition, cyclic voltammetry, nitrite oxidation

## Abstract

Nitrite (NO_2_^−^) has long been recognized as a contaminant of concern due to its detrimental effects on both human health and the environment. As a result, there is a continuing need to develop sensitive, real-time, low-cost, and portable systems for the accurate detection of trace levels of NO_2_^−^ in drinking water. We present a novel, low-cost, and easy-to-fabricate amperometric sensor designed for detecting low concentrations of NO_2_^−^ in drinking water. The fabrication technique involves the electrodeposition of manganese and copper oxides onto a carbon working electrode. CuO and MnO_2_ act synergistically as efficient catalysts for the electrooxidation of nitrite to nitrate (NO_3_^−^) thanks to their complementary redox properties. The resulting sensor exhibits high catalytic activity toward the electrooxidation of NO_2_^−^, with a sensitivity of 10.83 μA/µM, a limit of detection (LOD) of 0.071 µM, and a good linear dynamic concentration range (0.2–60 µM). The sensor’s performance was evaluated against potential interfering analytes (NO_3_^−^, Cl^−^, NH_4_^+^, and NH_2_Cl), all of which showed negligible interference. Reproducibility (maximum standard deviation 2.91%) and repeatability (usable up to three times) were also evaluated.

## 1. Introduction

The nitrite ion (NO_2_^−^) is a critical indicator of water quality due to its significant environmental and health implications [1,2]. It is widely used in food additives, such as those in cured meat products, chemical bleaches, soil fertilizers, and dying agents, as well as corrosion inhibitors. Additionally, it has some pharmaceutical uses, including cyanide poisoning antidotes and blood anticoagulation drugs [3]. Elevated nitrite concentrations in water can lead to eutrophication, toxicity, and the formation of carcinogenic *N*-nitroso compounds [4,5,6]. Furthermore, nitrite is associated with methemoglobinemia in infants, commonly referred to as “blue baby syndrome”, making its detection essential for public health monitoring [7,8]. Guidelines for nitrite intakes in drinking water were recommended by the World Health Organization (WHO), which has established an obligatory guideline referred to as the maximum contaminant level (MCL) for nitrite to be 43.48 µM, and 65.2 µM for drinking and raw water, respectively [9]. For this reason, efficient methods for detecting and removing nitrites from water are essential [10,11]. Electrochemical sensors have emerged as a promising solution for nitrite detection due to their simplicity, cost-effectiveness, and ability to operate in complex matrices [12]. Among these, screen-printed electrodes (SPEs) stand out for their portability, scalability, and ease of surface modification [13,14,15]. A screen-printed electrochemical cell is composed of three electrodes: the working electrode (WE), which may be functionalized with materials selective towards the analyte; the reference electrode (RE) to ensure the precise application of the WE potential; and the counter electrode (CE) to complete the circuit [16,17,18]. This system undergoes an electrochemical reaction that results in changes in current, potential, charge, or impedance, which are measured using different electrochemical techniques [19]. Several studies have demonstrated the potential of modified SPEs for nitrite detection, utilizing different electrode surface modifications [20,21,22,23]. Adiraju et al. developed an electrochemical sensor based on carbon SPE modified with gold nanoparticles for nitrite detection in groundwater, achieving a limit of detection (LOD) of 0.38 μM [24]. The principle of operation is the electrochemical oxidation of NO_2_^−^ at pH 6.5 in PBS buffer during the square wave voltametric (SWV) scan. Rajab et al. developed an electrochemical sensor based on SPE modified with zirconia nanoparticles and multi-walled carbon nanotubes (ZrO_2_@MWCNTs/SPE) for selective detection of nitrite in food and water samples. The SPE modification with composite ZrO_2_@MWCNTs resulted in enhanced peak current of nitrite compared to the bare electrode signal by enhancing the electron transfer rate. This is attributed to the combination of the tubular shape of MWCNTs and the crystallinity of the ZrO_2_, providing abundant electron transport pathways and active sites, which improves the nitrite absorption during the oxidation reaction. The main electrochemical reaction involved in the detection process is the oxidation of nitrite to nitrate. The device showed a LOD of 0.94 μM, with high sensitivity and selectivity [3].

This work presents an electrochemical sensor developed for monitoring nitrite ions in drinking water. Copper and manganese oxides were electrodeposited by cyclic voltammetry (CV) in a Carbon SPE [25,26,27]. CV is a powerful electrochemical technique used to study chemical reactions initiated by electron transfer, which includes catalysis [28,29,30]. The carbon electrode, chosen for its electrochemical stability and conductivity, provides an ideal substrate for the deposition of copper and manganese, further improving the process efficiency [31,32]. The choice of combining CuO and MnO_2_ for nitrite detection is motivated by their complementarity. While CuO has proven to be effective for nitrite in literature works [33,34,35] and in nitrate (NO_3_^−^) reduction in our previous work [22,31], MnO_2_ is widely recognized for its high activity towards nitrite oxidation. CuO and MnO_2_ work together synergistically as efficient catalysts for the electrooxidation of nitrite to nitrate, thanks to their complementary redox properties [36]. This study presents experimental results of the fabrication of an electrode sensitive to nitrite oxidation in water. Morphological and structural characterization was performed, and the electrocatalytic performance of the sensor in nitrite oxidation was described, analyzing the efficiency, selectivity, and stability of the process.

## 2. Materials and Methods

### 2.1. Chemicals and Equipment

Manganese Chloride (MnCl_2_), Copper sulphate pentahydrate (CuSO_4_·5H_2_O), sodium nitrite (NaNO_2_), Phosphate Buffered Saline (PBS), and potassium chloride (KCl) were purchased from Merck KGaA (Headquarters in Darmstadt, Germany) and used without further purification. Milli-Q water (resistivity of at least 18.2 MΩ cm) obtained by Simplicity UV (Millipore, by Merck, Headquarters in Darmstadt, Germany) was used in all solutions. SPCE (screen-printed carbon electrodes, cod. Ref. C110) were bought from Metrohm DropSens s.r.l. (Origgio, VA, Italy). They are a single-channel three-electrode system: the working electrode is manufactured with mesoporous carbon ink, the counter electrode with carbon ink, and the reference electrode with Ag/AgCl ink. CuO and MnO_2_ electrodeposition and all electrochemical measurements were performed by Palmsens4 electrochemical workstation by PalmSens BV (C-PS4-BP.F2.10, GA Houten, The Netherlands). Raman analyses were performed using a Horiba Labram HR Evolution spectrometer, adopting a 532 nm laser and a grating at 1800 cm^−1^, to achieve high resolution. Scanning electron microscopy (SEM) images were obtained using a ZEISS FE-SEM SUPRA 35 (Carl Zeiss AG, Jena, Germany). X-ray Photoelectron Spectroscopy (XPS) analysis was carried out using a PHI Genesis Multi-Technique Scanning XPS system, with a monochromatic Al Kα (1486.6 eV) X-ray beam and a 180° hemispherical electron energy analyzer. X-ray diffraction patterns were collected using SmartLab equipment made by Rigaku (Tokyo, Japan).

### 2.2. Working Electrode Functionalization

The Mn particles were deposited electrochemically on the mesoporous carbon WE surface (4-mm diameter) by cyclic voltammetry (CV). The potential ranged from 0.0 V to 1.2 V at a scan rate of 0.05 V s^−1^. Five scans were performed using 0.1 M MnCl_2_ in 1 M KCl supporting electrolyte. The electrodeposition of MnO_2_ occurred via an anodic process, where Mn^2^^+^ ions were oxidized to Mn^4^^+^ under the application of anodic potentials. The overall reaction is: Mn^2^^+^ + 2H_2_O → MnO_2_ + 4H^+^ + 2e^−^.

Mn^2^^+^ ions adsorb onto the carbon surface. At moderate potentials, Mn^2^^+^ is oxidized to the intermediate Mn^3^^+^and subsequently is further oxidized to Mn^4^^+^, thus precipitating as hydrated MnO_2_. This process explains why the XPS analyses found traces of Mn_2_O_3_ oxides.

Subsequently, Cu particles were electrodeposited via CV in the potential range from −1.0 V to 0.0 V at a scan rate of 0.05 V s^−1^ by scanning 5 times using 0.1 M CuSO_4_∙5H_2_O in 1 M KCl supporting electrolyte [21]. The presence of MnO_2_ on the electrode surface promotes the formation of CuO rather than metallic Cu because, under anodic polarization, MnO_2_ remains stable and may even participate in redox mediation.

### 2.3. Electrode Performance Analysis

The performance of the developed electrode was evaluated using Linear Sweep Voltammetry (LSV). The potential was scanned from 0.5 V to 1.1 V at a scan rate of 0.05 V s^−^^1^. LSV measurements were conducted at various concentrations of NO_2_^−^ (0.2–60 µM) in a 0.01 M PBS electrolyte solution (pH 7).

## 3. Results

### 3.1. Structural Characterization

**Figure 1:**
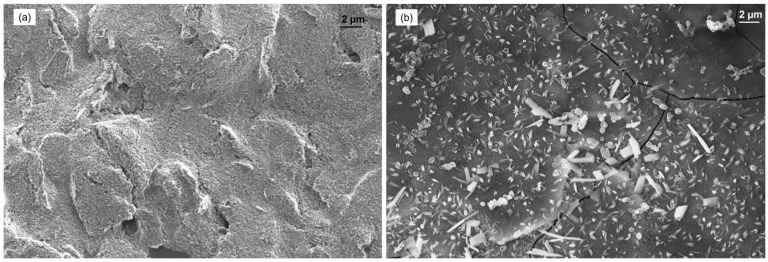
SEM images of: carbon bare electrode (**a**), Cu-Mn/C modified electrode (**b**).

Scanning electron micrographs were acquired to study the Carbon surface, the electrodeposited manganese and copper morphologies (Figure 1). The bare carbon electrode (Figure 1a) exhibits a relatively uniform surface with characteristic microporous structure and shallow fissures typical of screen-printed carbon matrices. In contrast, the Cu-Mn/C modified electrode (Figure 1b) shows a different morphology characterized by a dense distribution of rod-like and needle-shaped nanocrystallites (100–500 nm in length) heterogeneously dispersed across the carbon substrate. The Energy-Dispersive X-ray spectroscopy (EDX) analysis spectrum (Figure S1) confirms the successful co-deposition of manganese and copper species, with quantitative analysis revealing a predominance of copper (45.20 wt%) relative to manganese (4.29 wt%). The significant oxygen content (29.39 wt%) corroborates the formation of metal oxides rather than elemental metals, while residual chlorine (9.19 wt%) likely originates from the KCl supporting electrolyte employed during the electrodeposition process.

**Figure 2:**
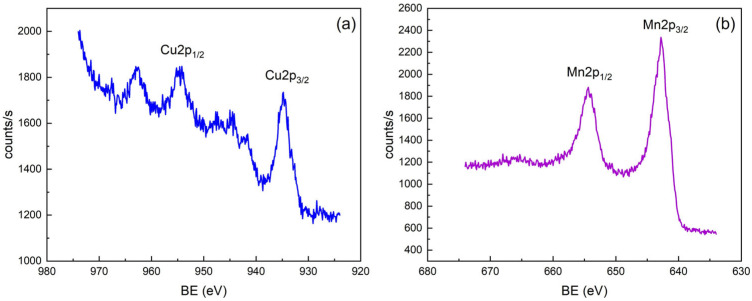
XPS spectra of Cu-Mn/C electrode: Cu (**a**), Mn (**b**).

Surface modification of carbon electrode with manganese and copper was characterized using Raman and X-ray photoelectron spectroscopy (XPS). Raman analysis (Figure S2) reveals distinct peaks in the 0–800 cm^−1^ region, with a sharp band at 150 cm^−1^ attributed to Mn-O-Mn bridging vibrations in MnO_2_ [37,38]. The peaks at 200–250 cm^−1^ correspond to overlapping modes of Mn_3_O_4_/Mn_2_O_3_ and CuO, while the wide feature between 500–700 cm^−1^ is due to Mn-O stretching in MnO_2_ (570–650 cm^−1^) and CuO (615 cm^−1^) [39,40,41]. XPS analysis confirms the results obtained by Raman analysis. The Cu 2p region shows a Cu 2p_3/2_ peak at 933 eV with a characteristic satellite at 940–945 eV, confirming the presence of Cu(II) in CuO (Figure 2a) [42,43]. The Mn 2p spectrum shows a Mn 2p_3/2_ peak at 641 eV, consistent with Mn(IV) in MnO_2_(Figure 2b) [44,45,46]. The survey scan (Figure S3) confirms the presence of C 1s (284 eV), O 1s (530 eV).

The electrode surface comprises a heterogeneous layer of MnO_2_ and CuO, with potential minor contributions from Mn_2_O_3_ and Cu(OH)_2_. Due to the mixed redox-active sites, this bifunctional oxide coating, validated by vibrational and electronic structure analyses, is ideal for electrocatalytic applications [47].

XPS analysis reveals a significant decrease in Cu and Mn signal intensities after nitrite detection, compared to the pristine Cu-Mn/C electrode. Initially, Cu 2p and Mn 2p spectra confirm the presence of CuO and MnO_2_, respectively. Following the sensing reaction, the marked reduction in these peaks indicates a loss or transformation of the metal oxides. This suggests that CuO and MnO_2_ actively participate in the electrochemical detection of nitrite through redox processes, leading to their partial reduction, dissolution, or structural modification. Such changes highlight the involvement of these oxides as active catalytic sites, consistent with the typical behavior of transition metal oxide-based sensors (Figure S4).

The presence of CuO and MnO_2_ on the carbon electrode, confirmed by XPS and Raman spectroscopy, was not detected by X-ray diffraction analysis (XRD). This suggests that these metal oxides are primarily present in poorly crystalline or amorphous phases, which lack long-range order and/or are highly dispersed on the carbon substrate, preventing the formation of sufficiently large crystalline domains. Therefore, the heterogeneous nature of the deposit, characterized by aggregates of different sizes and morphologies, suggests the formation of multiple oxide phases.

### 3.2. Electrochemical Characterization

**Figure 3:**
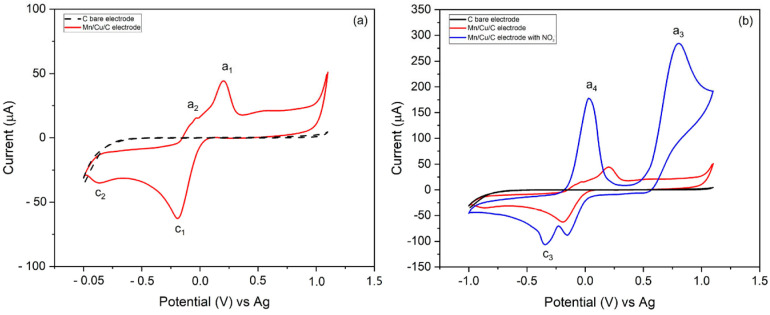
Cyclic voltammograms in 0.01 M PBS supporting electrolyte of: bare C electrode (black trace) and Cu-Mn/C modified electrode (red trace) (**a**); bare C electrode (black trace), Cu-Mn/C modified electrode (red trace) and Cu-Mn/C modified electrode in the presence of 30 µM NO_2_^−^ (blue trace) (**b**).

Electrochemical characterization of the bare carbon electrode (SPCE) and Cu-Mn/C modified electrodes was performed in a 0.01 M PBS electrolyte solution, in the absence and presence of nitrite concentration (30 µM NO_2_^−^) (Figure 3a,b). SPCE (black trace) exhibits featureless behavior in the investigated potential windows, confirming the electrochemical inertness of the electrode both in the absence and presence of nitrite ions. The modified Cu-Mn/C electrode (red trace) exhibits a cathodic peak c_1_ at −0.3 V, which likely corresponds to the reduction of copper oxides (CuO to Cu^0^), and an anodic peak a_1_ at +0.2 V corresponding to the oxidation of Cu^0^ back to CuO [48,49]. The peak separation (ΔEp) between reduction and oxidation is significant (0.4 V), indicating that the redox process is irreversible. The anodic peak a_2_ at +0.0 V is due to the reduction of manganese oxides (MnO_2_ to Mn^2+^), and the cathodic peak c_2_ at −0.7 V is the reverse process [48,49,50].

In the presence of nitrite (blue trace in Figure 3b), the anodic peak present at approximately a_3_ +0.8 V is attributable to the oxidation of nitrite to nitrate [27,28,51]. The cathodic peak c_3_ at −0.4 V may be related to the reduction of nitrate to nitrite or other products. Furthermore, the voltammogram of the modified electrode shows a significant enhancement and overlapping of the CuO and MnO_2_ reduction peaks (a_4_), which are also shifted towards a more negative potential. This behavior indicates that the metal oxides act as electrocatalysts for NO_2_^−^ oxidation. The electrocatalytic mechanism involves CuO and manganese MnO_2_ species on the electrode surface, which work synergistically to reduce the activation energy required for nitrite oxidation to nitrate.

**Figure 4:**
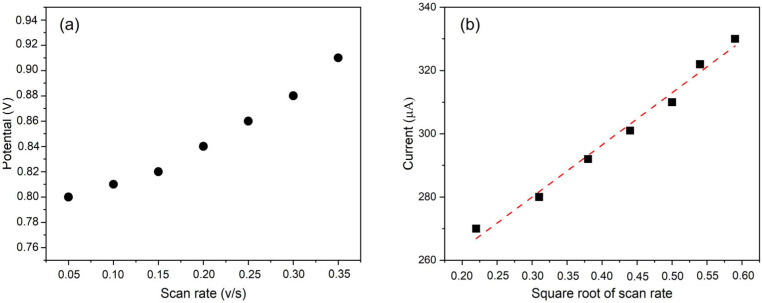
The NO_2_^−^ oxidation peak potential as a function of the scan rate (**a**). Maximum peak current as a function of the square root of the scan rate. The dashed red line is the linear best fit of the data (**b**).

To evaluate the nature of the NO_2_^−^ electrochemical reaction at the Cu-Mn/C electrode, the effect of the scan rate (50 to 350 mV/s) on the oxidation peak current was investigated. The anodic peak current (*i_p_*) increased linearly with the square root of the scan rate (ν^1^ᐟ^2^), as shown in Figure 4b. This behavior is characteristic of a diffusion-controlled electrochemical process, in agreement with the Randles–Ševčík equation for irreversible systems: [52]

(1) $$i_{p}=\left(2.99\times 10^{5}\right)*n[\alpha {n_{a}}^{\frac{1}{2}}]{AD}_{0}^{\frac{1}{2}}{C\nu }^{\frac{1}{2}}$$

where *i_p_* is the peak current, α is the transfer coefficient and *n_a_* is the number of electrons involved in the rate-determining step (2 for NO_2_^−^), *A* is the active surface area (cm^2^), *D*_0_ is the diffusion coefficient (1.9 × 10^−5^ cm^2^ s^−1^ for NO_2_^−^ at 25 °C), v is the scan rate (V s^−1^), and C is the NO_2_^–^ concentration (mol cm^–3^) [3].

The observed significant peak separation (Figure 3b) and the shift in peak potentials with increasing scan rate further confirm the irreversible nature of the NO_2_^−^ oxidation process at the Cu-Mn/C electrode. The Randles–Ševčík equation for irreversible systems was used to interpret the data, as the process displays significant peak separation and asymmetry, the redox process is electrochemically irreversible [53].

### 3.3. Sensor Performance for Nitrite Ion Detection

**Figure 5:**
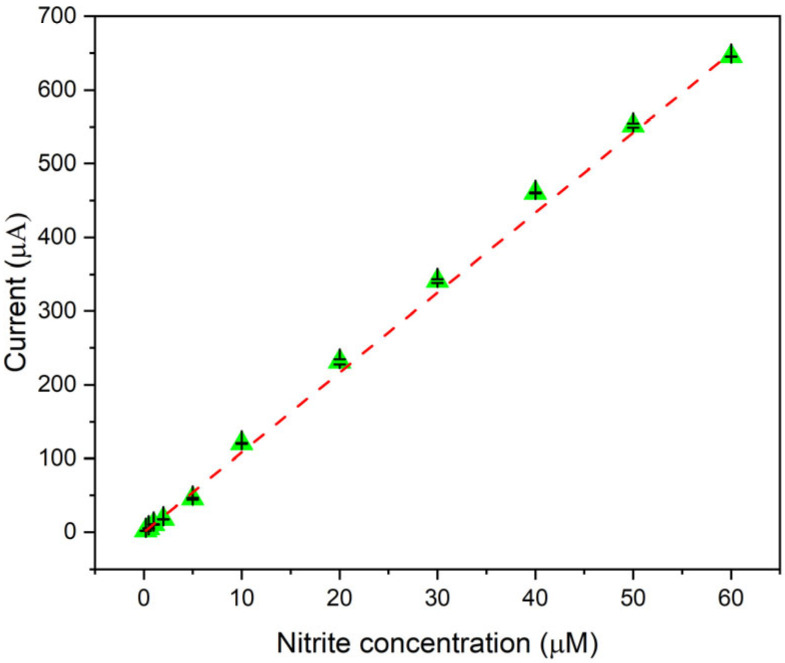
Calibration curve of the electrode as a function of the nitrite concentration (i.e., 0.2, 0.5, 1, 2, 5, 10, 20, 30, 40, 50, and 60 µM). The points, representing the average peak current measured by three sensors, indicate the anodic current peak maximum at 0.8 V. The SD is represented by the error bars.

Preliminary studies were conducted to evaluate the performance of the electrode modified exclusively with MnO_2_ for the detection of nitrite ions in water (Figure S5). The results revealed a non-linear response in the detection performance for the nitrite ion. This observation further motivated the incorporation of Cu onto the Mn/C-based electrode to enhance the sensor performance. To evaluate the analytical performance of the final Cu-Mn/C developed sensor, Linear Sweep Voltammetry (LSV) was employed at different concentrations of NO_2_^−^ in 0.01 M PBS electrolyte solution (pH = 7), (Figure S6). The calibration curve was obtained by averaging the NO_2_^−^ reduction peak current of three samples at each of the concentration measured (i.e., 0.2, 0.5, 1, 2, 5, 10, 20, 30, 40, 50, and 60 µM) with the standard deviation (SD) (Figure 5). The data showed a linear detection range from 0.2 to 60 µM with a sensitivity of 10.83 μA/µM and a coefficient of determination (*R*^2^) of 99.87%, indicating an excellent linear fit (Figure S7). Based on a signal-to-noise ratio of three, the LoD was estimated to be 0.071 µM (71 nM). The comparison with Figure S5 (electrode modified with only MnO_2_) clearly explains the need to use two metals to obtain a performing sensor.

The choice of PBS (pH 7) as the supporting electrolyte was made to provide a stable and well-controlled environment, advantageous for consistent and reproducible electrochemical measurements. Although PBS is commonly used in biological and physiological studies, its neutral pH and buffering capacity make it a suitable medium to simulate natural water conditions in environmental monitoring, where pH values often approach neutrality. On the other hand, this study paves the way for the use of the nitrite sensor for applications in physiological samples or sanitized waters.

The nitrite detection performance observed with our sensor appears promising in comparison with recent work based on the manufacturing of SPE-based electrochemical sensors for nitrite detection (Table 1) [3,24,26,54].

The developed electrode, based on SPE modified with CuO and MnO_2_, exhibits a lower limit of detection of 71 nM, which is more sensitive than most previously reported sensors. Although a comparable LoD of 74 nM was achieved using a Cu-BDC/Fe_2_O_3_-modified SPCE [26], the sensor developed in this work provides a wide linear detection range of 0.2–60 µM, which is particularly suitable for monitoring nitrite levels in drinking water, where regulatory limits are typically in the low micromolar range. In contrast, other reported sensors exhibit higher LoDs (0.38–1.98 µM) and lower limits of linear ranges (5–10 µM) [3,24,55] limiting their applicability for trace analysis. Additionally, the use of non-toxic and cost-effective CuO and MnO_2_ nanomaterials in electrode modification enhances the environmental friendliness and practical feasibility of the proposed sensor. These findings confirm that the developed sensor provides a highly sensitive, selective, and practical approach for the reliable determination of nitrite in drinking water samples.

### 3.4. Reproducibility and Repeatability of the Sensor

The reproducibility and repeatability of the sensor were evaluated. Reproducibility refers to the consistency of sensor responses when measurements are performed using different electrodes fabricated under the same conditions. Repeatability refers to the consistency of measurements obtained using the same electrode over multiple trials, reflecting the sensor’s stability and performance over time. Both aspects are critical since reproducibility ensures that the sensor fabrication process is reliable, while repeatability indicates the sensor’s operational stability during use. The first was assessed by measuring the same solution with three different electrodes, all made following the same procedure. In contrast, repeatability was evaluated by performing multiple measurements on the same solution with the same electrode. For the reproducibility test, eleven different nitrite concentrations (i.e., 0.2, 0.5, 1, 2, 5, 10, 20, 30, 40, 50, and 60 µM) were tested, and each concentration was measured three times. The relative standard deviations (RSDs) were 6.48%, 3.74%, 4.77%, 1.49%, 2.91%, 0.43%, 1.39%, 0.78%, 0.13%, 0.52%, and 0.04%, respectively, confirming good reproducibility (Figure S8). This result highlights the reliability of the electrode functionalization process. For the repeatability test, five consecutive measurements were carried out using the same electrode in a 30 µM NO_2_^−^ solution. The sensor maintained stable performance up to the third measurement, which showed a 6.5% decrease in the anodic peak current compared to the first measurement (Figure S9). This indicates that while the sensor can be reused, its performance gradually diminishes with repeated use. Since screen-printed electrodes (SPEs) are generally designed as disposable devices, our sensor shows promising reusability for a limited number of measurements, which could be advantageous in applications requiring multiple analyses without electrode replacement. Storage conditions for the electrodes involve keeping them in a dry, dark environment at room temperature when not in use to minimize degradation. The electrodes were rinsed with deionized water between measurements to remove residual analyte and dried gently with nitrogen gas. No additional regeneration steps were applied, which may contribute to the observed signal decrease upon reuse.

### 3.5. Interference Study

The Cu-Mn/C modified electrode exhibited exceptional selectivity for nitrite (NO_2_^−^) in the presence of common interferents, including nitrate (NO_3_^−^), ammonium (NH_4_^+^), and monochloramine (NH_2_Cl). Cyclic voltammetry confirmed the absence of redox activity for these species within the sensor’s operational window (0.6–1.0 V vs. Ag), with no detectable oxidation peak observed in nitrite-free solutions containing the interferents. Since the LoD of the sensor is very low, it was decided to use interferent concentrations (30 µM) equal to that of nitrite (30 µM) for interference measurements (Figure S10). This approach allows a direct and comparable evaluation of the interferent’s effect at the same concentration as the analyte, enabling the study of selectivity under controlled conditions. In the presence of interferents and nitrite, the anodic peak current remained stable, demonstrating robust selectivity. It is due to the electrocatalytic properties of the MnO_2_/CuO interface. In addition, the sensor was used in another work (N. Marino et al.) [55] to make quantitative measurements on an electrolytic solution for ammonia production in which nitrate, ammonium ions, and other reduced nitrogen species (such as NH_2_OH and N_2_H_4_) were present. Also, there was no interference with the measurement of nitrite concentration. The nitrite detection results matched the calibration curve obtained in PBS (R^2^ = 0.997), confirming the sensor’s accuracy even in complex matrices. This is because, although NO_3_^−^ is structurally similar to NO_2_^−^, it requires a higher oxidation potential (+1.2 V vs. Ag) compared to NO_2_^−^ (+0.8 V), which prevents it from undergoing electron transfer under the same conditions. NH_4_^+^ has no accessible redox states at the applied potential and therefore remains electrochemically inactive. The MnO_2_/CuO synergy, combining Mn^4^^+^/Mn^3^^+^ redox mediation with CuO’s oxygen-vacancy-enabled adsorption, effectively discriminates against non-target species while stabilizing NO_2_^−^ intermediates. This dual mechanism ensures precise nitrite detection in industrial and environmental applications, even in the presence of competing nitrogen species.

## 4. Conclusions

This work presents a novel, cost-effective, and easily manufactured amperometric sensor for nitrite detection in drinking water. The sensor is based on the electrodeposition of manganese and copper oxides onto a carbon screen-printed electrode. The synergistic catalytic activity of CuO and MnO_2_ enables highly sensitive nitrite detection with a sensitivity of 10.83 μA/µM, a low limit of detection of 0.071 µM, and a wide linear dynamic range from 0.2 to 60 µM. The sensor exhibits insignificant interference from common water contaminants, excellent reproducibility, and acceptable repeatability for multiple measurements. These features, combined with its ease of fabrication, make it a promising candidate for real-time, on-site monitoring of nitrite levels in environmental and industrial applications. Further research will focus on long-term stability, miniaturization, and integration into portable devices for widespread use.

## Figures and Tables

**Table 1:** Comparison of this work with some of the literature electrochemical sensing platforms for nitrite detection.

Electrode	LoD	Linear Range	Application	Reference
SPE modified with CuO and MnO_2_ (This work)	71 nM	0.2–60 µM	Drinking water	This work
SPCE modified with copper(II)-benzene-1,4-dicarboxylate (Cu-BDC) frameworks and Fe_2_O_3_ NPs	74 nM	1–2000 µM	Mineral water	[26]
AuNP-modified screen-printed carbon electrode (SPCE)	0.38 µM	5.0–100 µM	On-site detection in aqueous samples	[24]
SPE modified with ZrO_2_@MWCNTs	0.94 µM	5.0–100 µM	Food and water samples	[3]
Polydopamine/AuNPs-modified SPCE	1.98 μM	10 to 500 μM	Processed meat samples in water	[54]

## Data Availability

The raw data supporting the conclusions of this article are included in the paper and Supplementary Materials figures and tables.

## Supplementary Materials

The following supporting information can be downloaded at: https://www.mdpi.com/article/10.3390/s25123764/s1, Figure S1: EDX analysis of Cu-Mn/C electrode, Figure S2: Raman spectra of Cu-Mn/C electrode, Figure S3: XPS spectra of Cu-Mn/C electrode, Figure S4: XPS spectra of Cu-Mn/C electrode after the use, Figure S5: Calibration curve of the electrode as a function of the nitrite concentration (i.e., 20, 30, 40, 50, and 60 µM), Figure S6: LSV measurement at nitrite concentration of 0.2, 0.5, 1, 2, 5, 10, 20, 30, 40, 50, and 60 µM, Figure S7: Calibration curve of the electrode as a function of the nitrite concentration (i.e., 0.2, 0.5, 1, 2, 5, 10, 20, 30, 40, 50, and 60 µM). The points, representing the average peak current measured by three sensors, indicate the anodic current peak maximum at 0.8 V. The SD is represented by the error bars, Figure S8: Reproducibility test for Cu-Mn/C electrode, Figure S9: Repeatability test for Cu-Mn/C electrode, Figure S10: Interfering tests.
