# Supplementary material for: Electrocatalytical Nitrite Oxidation via Manganese and Copper Oxides on Carbon Screen-Printed Electrode
Source: Sensors (Basel). 2025 Jun 16;25(12):3764. doi: 10.3390/s25123764 (PMC12197279; doi:10.3390/s25123764)
Supplement: Supplementary file 1 [file sensors-25-03764-s001.zip › sensors-3667077-supplementary.pdf]

SUPPLEMENTARY MATERIALS

Electrocatalytical Nitrite Oxidation via Manganese and Copper Oxides on Carbon Screen-Printed Electrode

Roberta Farina [1,2,*], Silvia Scalese [1], Alessandra Alberti [1], Stefania Maria Serena Privitera [1], Giuseppe Emanuele Capuano [1], Domenico Corso [1,3], Giuseppe Andrea Screpis [1,4,5], Serena Concetta Rita Reina [6], Guglielmo Guido Condorelli [2], Maria Anna Coniglio [1,6] and Sebania Libertino [1,*]

AUTHOR ADDRESS

1 Consiglio Nazionale delle Ricerche—Istituto per la Microelettronica e Microsistemi (CNR-IMM), Strada VIII Z.I., 5, 95121 Catania, Italy

2 Dipartimento di Scienze Chimiche, Università Degli Studi di Catania, Viale A. Doria 6, 95125 Catania, Italy

3 Department of Electrical, Computer and Biomedical Engineering, Università Degli Studi di Pavia, Via Ferrata 5, 27100 Pavia, Italy

4 Dipartimento di Scienze Chimiche, Biologiche, Farmaceutiche e Analitiche (ChiBioFarAm), Università Degli Studi di Messina, Viale F. Stagno d'Alcontres 31, Vill. S. Agata, 98166 Messina, Italy

5 Consiglio Nazionale delle Ricerche—Istituto per lo Studio dei Materiali Nanostrutturati, CNR-ISMN, Viale F. Stagno d'Alcontres 31, Vill. S. Agata, 98166 Messina, Italy

6 Dipartimento di Scienze Mediche, Chirurgiche e Tecnologie Avanzate "G.F. Ingrassia", Università Degli Studi di Catania, Via S. Sofia 87, 95123 Catania, Italy

Morphological and Compositional Characterization

The EDX spectrum (Figure S1) confirms the successful co-deposition of manganese and copper species, with quantitative analysis revealing a predominance of copper (45.20 wt%) relative to manganese (4.29 wt%). The significant oxygen content (29.39 wt%) corroborates the formation of metal oxides rather than elemental metals, while residual chloride (9.19 wt%) likely originates from the KCl supporting electrolyte employed during the electrodeposition process.

The elemental mapping images of the carbon electrode modified with CuO and MnO_2 provide clear evidence of the spatial distribution of copper, manganese, and carbon on the electrode surface. The Cu L map shows a highly uniform and dense distribution of copper species across the entire analysed area, indicating a homogeneous coverage of CuO on the electrode surface. The Mn K map reveals that manganese is also well-dispersed throughout the surface, though with a slightly less dense distribution compared to copper. The presence of MnO_2 is confirmed by the widespread but discrete magenta spots, suggesting that MnO_2 is effectively deposited and accessible for electrocatalytic reactions. The C K map illustrates the underlying carbon substrate, which appears as a relatively sparse but evenly distributed signal. This confirms that the carbon electrode remains exposed and accessible, providing electrical conductivity and mechanical support for the metal oxide layers.

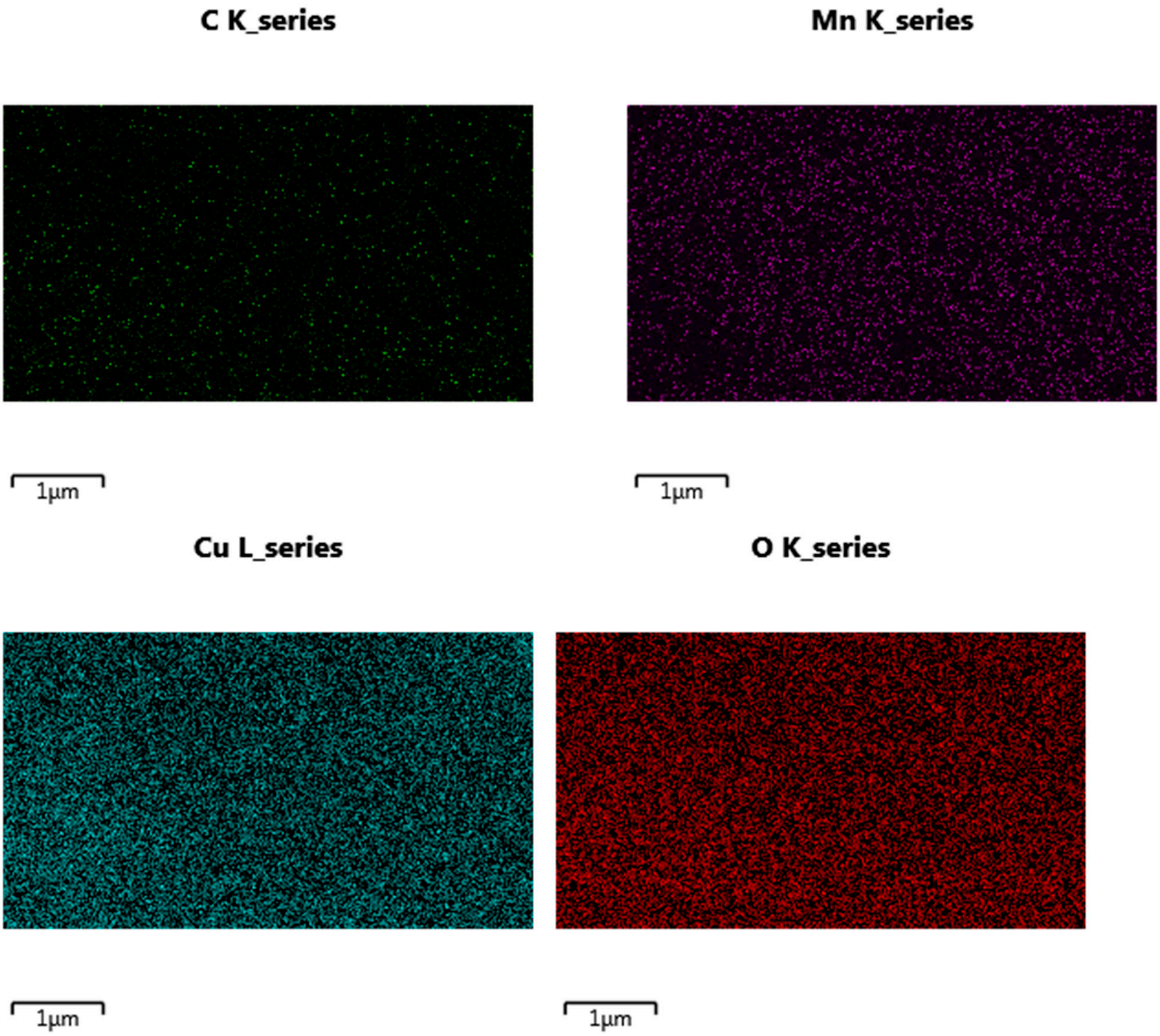

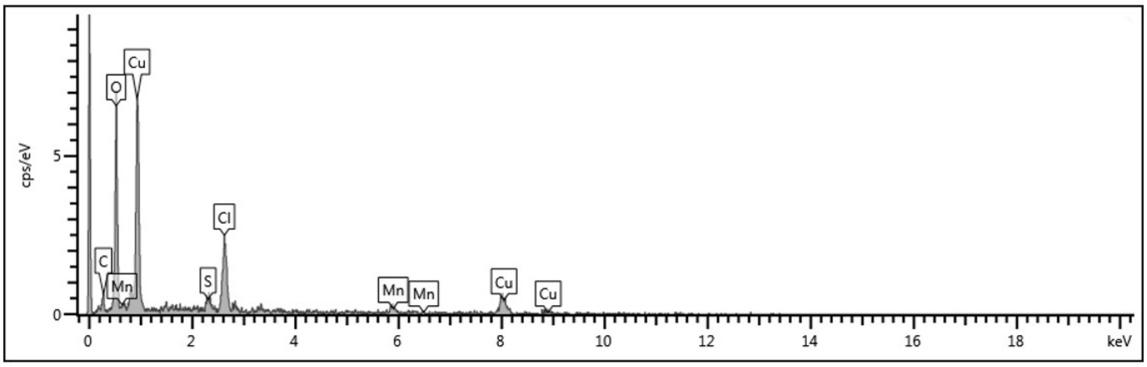

Element	Wt %	Wt% Sigma
C	10.45	2.24
O	29.39	1.43
S	1.48	0.34
Cl	9.19	0.67
Mn	4.29	0.85
Cu	45.20	1.83
Total:	100.00	

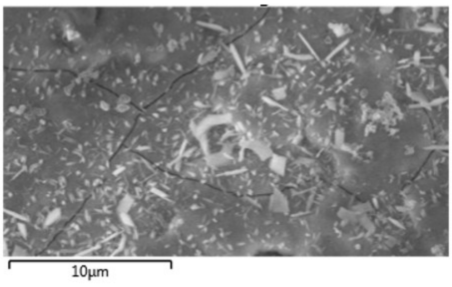

Figure S1. EDX analysis of Cu-Mn/C electrode

Raman analysis (Figure S2) reveals distinct peaks in the 0-800 cm^{-1} region, with a sharp band at 150 cm^{-1} attributed to Mn-O-Mn bridging vibrations in MnO_2. The peaks at 200-250 cm^{-1} correspond to overlapping modes of Mn_3O_4/Mn_2O_3 and CuO, while the wide feature between 500-700 cm^{-1} comes from Mn-O stretching in MnO_2 (570-650 cm^{-1}) and CuO (615 cm^{-1}).

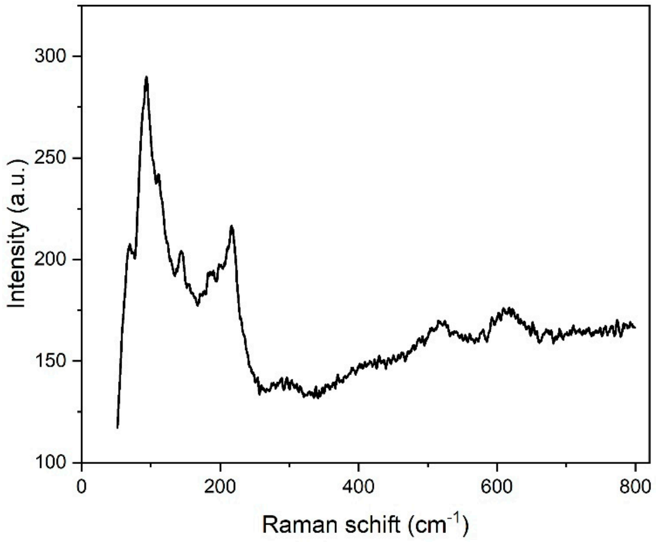

Figure S2. Raman spectra of Cu-Mn/C electrode

In XPS analysis, the Cu 2p region shows a Cu $2p_{3/2}$ peak at 933 eV with a characteristic satellite at 940-945 eV, confirming the presence of Cu(II) in CuO (Figure 2a). The Mn 2p spectrum shows a Mn $2p_{3/2}$ peak at 641 eV, consistent with Mn(IV) in MnO_2, although small shoulders suggest traces of Mn(III) species (Figure 2b). The survey scan (Figure S3) confirms the presence of C 1s (285 eV), O 1s (530 eV).

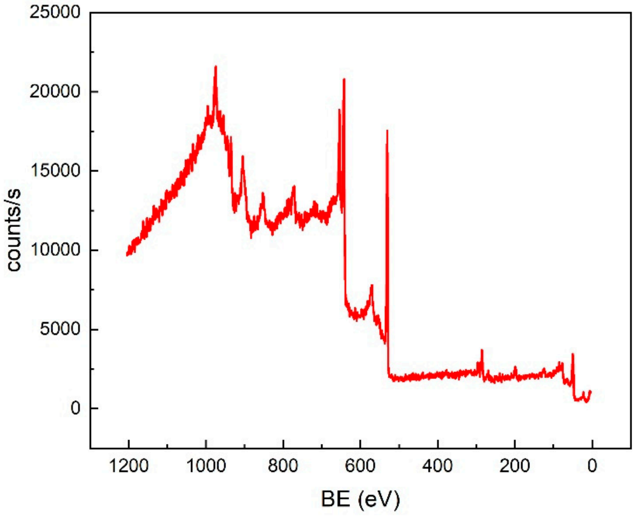

Figure S3. XPS spectra of Cu-Mn/C electrode

XPS analysis reveals a significant decrease in Cu and Mn signal intensities after nitrite detection, compared to the pristine Cu-Mn/C electrode. Initially, Cu 2p and Mn 2p spectra confirm the presence of CuO and MnO_2, respectively. Following the sensing reaction, the marked reduction in these peaks indicates a loss or transformation of the metal oxides. This suggests that CuO and MnO_2 actively participate in the electrochemical detection of nitrite through redox processes, leading to their partial reduction, dissolution, or structural modification. Such changes highlight the involvement of these oxides as active catalytic sites, consistent with the typical behavior of transition metal oxide-based sensors (Figure S4).

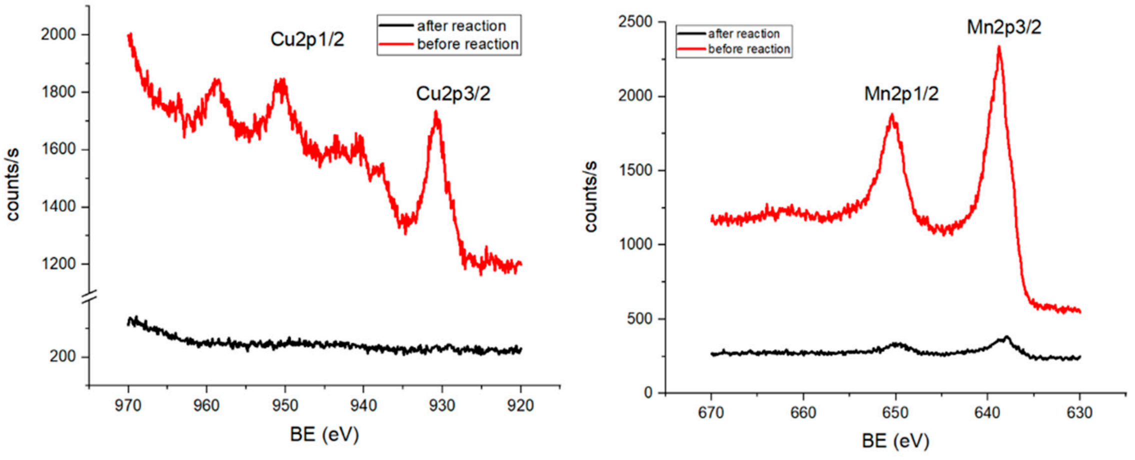

Figure S4. XPS spectra of the Cu-Mn/C fabricated electrode in the Cu and Mn regions, before (red line) and after (black line) use.

Electrochemical Characterization

Measurements were carried out using the electrode modified only with MnO_2, using the same procedure for electrode preparation described in the Materials and Methods section. The results revealed a poorer response (R2 = 0.9892) in the detection performance for the nitrite ion (Figure S5) compared to the Cu-Mn/C modified electrode (R2 = 0.9987). The senor has a LoD of 0.097 μM worse than that of the Cu-Mn/C sensor. This observation further motivated the incorporation of Cu onto the Mn/C-based electrode to enhance the performance of MnO_2 alone. Indeed, after this modification, excellent linearity in the detection response was achieved, which enabled a significant reduction in the detectable concentration range of the nitrite ions.

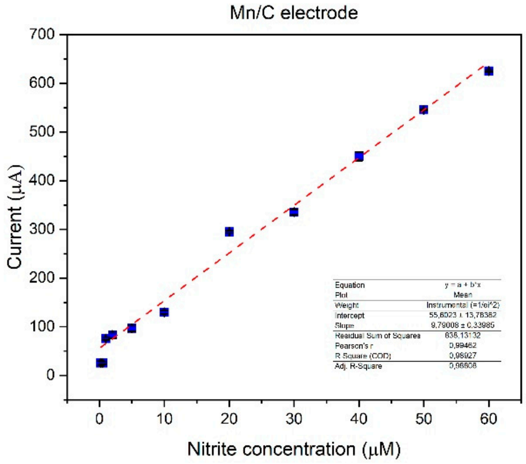

Figure S5. Calibration curve of the carbon electrode modified with MnO_2 particles (Mn/C electrode) as a function of the nitrite concentration (i.e. 0.2, 0.5, 1, 2, 5, 10, 20, 30, 40, 50, and 60 μM).

To evaluate the analytical performance of the sensor, Linear Sweep Voltammetry (LSV) was employed with different concentrations of NO_2^- (0.2, 0.5, 0.1, 2, 5, 10, 20, 30, 40, 50, and 60 μM) in 0.01 M PBS electrolyte solution (pH=7), (Figure S6). In the analysis, the peak was followed at +0.8 V.

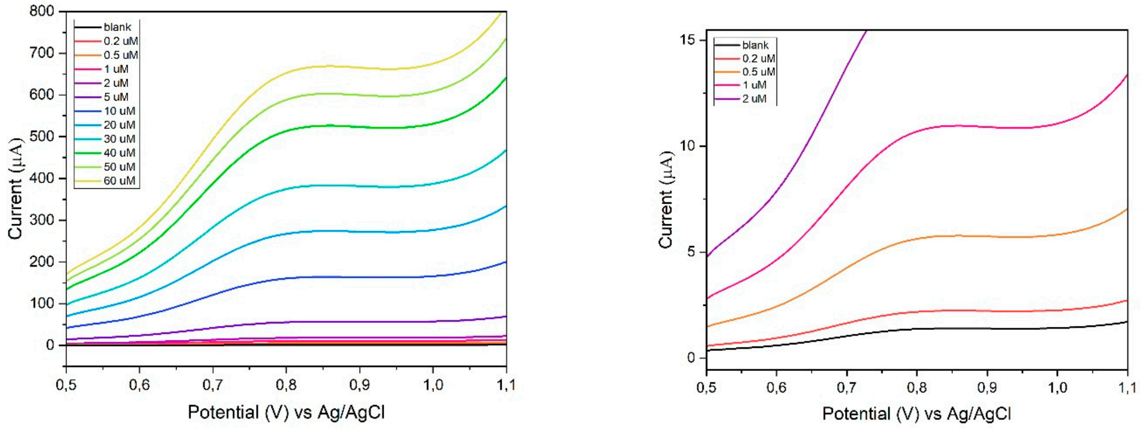

Figure S6. LSV measurement at nitrite concentrations of 0.2, 0.5, 1, 2, 5, 10, 20, 30, 40, 50, and 60 μM.

The calibration curve was obtained by averaging the NO_2^- reduction peak current of three samples at every concentration measured (i.e., 0.2, 0.5, 1, 2, 5, 10, 20, 30, 40, 50, and 60 μM) with the standard deviation (SD). The data showed a linear detection range from 0.2 to 60 μM with a sensitivity of 10.83 μA/μM and a coefficient of determination (R^2) of 99.87%, indicating an excellent linear fit (Figure S7).

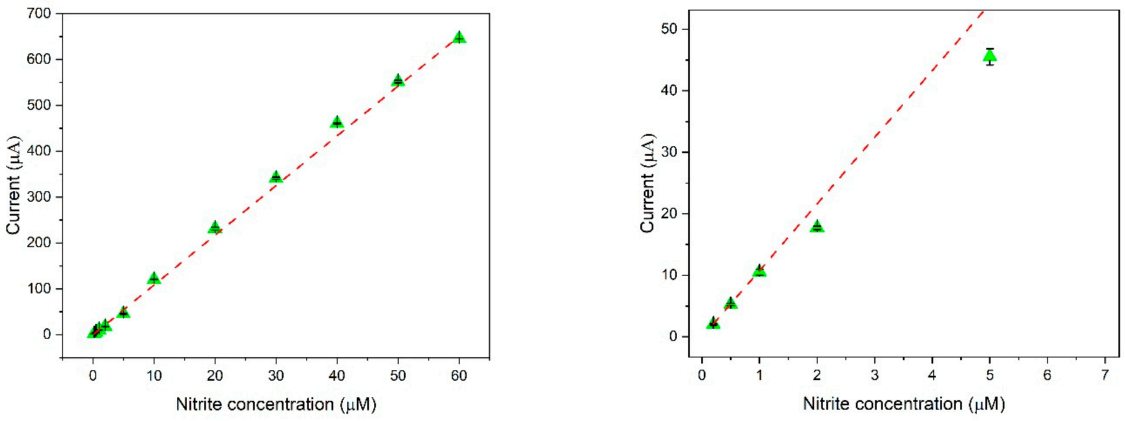

Figure S7. Calibration curve of the electrode as a function of the nitrite concentration (i.e., 0.2, 0.5, 1, 2, 5, 10, 20, 30, 40, 50, and 60 μM). The points, representing the average peak current measured by three sensors, indicate the anodic current peak maximum at 0.8 V. The SD is represented by the error bars.

Reproducibility and Repeatability of the Sensor

In the reproducibility test, eleven different nitrite concentrations (0.2, 0.5, 1, 2, 5, 10, 20, 30, 40, 50, and 60 μM) were tested, and each concentration was measured three times. The relative standard deviations (RSDs) were 6.48 %, 3.74 %, 4.77 %, 1.49 %, 2.91 %, 0.43 %, 1.39 %, 0.78 %, 0.13 %, 0.52 %, 0.04% respectively, confirming good reproducibility (Figure S8). This result highlights the reliability of the electrode functionalization process.

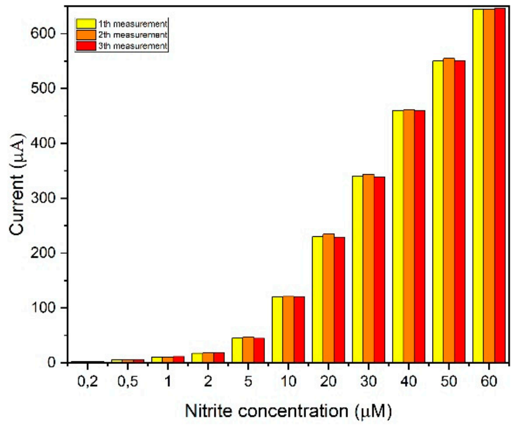

Figure S8. Reproducibility test for Cu-Mn/C electrode

For the repeatability test, five consecutive measurements were carried out using the same electrode in a 30 μM NO_2^- solution. The sensor maintained stable performance up to the third measurement,

which showed a 6.5% decrease in the anodic peak current compared to the first measurement (Figure S9).

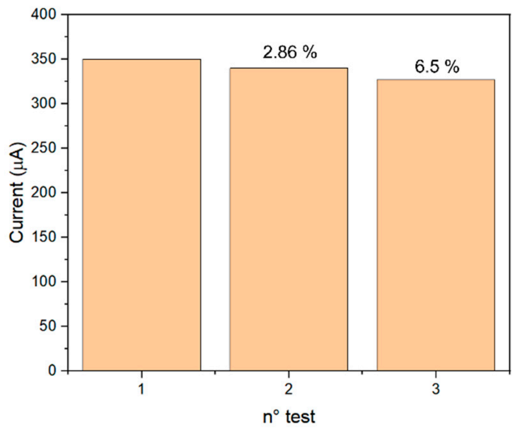

Figure S9. Repeatability test for Cu-Mn/C electrode

In the presence of nitrate ions, ammonium ions, and monochloramine, the voltammogram of the electrode does not exhibit any oxidation peaks at 0.8 V. Furthermore, the voltammogram is nearly identical to that obtained in the presence of the supporting electrolyte alone. Detailed linear sweep voltammetry measurements were conducted, focusing on the current values at 0.8 V for individual solutions containing the potential interferents. The results indicate that these species do not interfere significantly, as the current responses at a concentration of 30 μM deviate only slightly from the blank measurement (Figure S10).

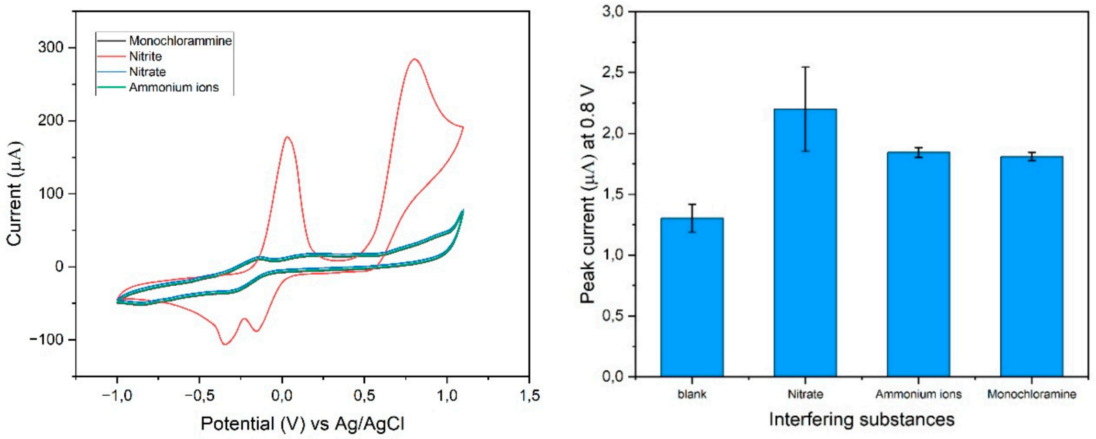

Figure S10. Interference of reduction peak current at 0.8 V in the presence of nitrate ions, ammonium ions, and monochloramine, all of them at a concentration of 30 μM.
